# MicroRNA-181a-5p Promotes Osteosarcoma Progression via PTEN/AKT Pathway

**DOI:** 10.1155/2022/3421600

**Published:** 2022-03-08

**Authors:** Chen Sun, Chi Chen, Zhen Chen, Jun Guo, Zhi-Hong Yu, Wei Qian, Fen Ai, Liang Xiao, Xiao Guo

**Affiliations:** ^1^Department of Orthopedic Surgery, Renmin Hospital, Hubei University of Medicine, Shiyan, Hubei, China; ^2^Department of Emergency, The Central Hospital of Wuhan, Tongji Medical College, Huazhong University of Science and Technology, Wuhan, Hubei, China

## Abstract

Osteosarcoma is the most common primary malignant bone tumor in children and adolescents with poor prognosis. MicroRNA-181a-5p (miR-181a-5p) is involved in the progression of various tumors; however, its role and underlying mechanism in osteosarcoma remains unclear. In this study, we found that miR-181a-5p was upregulated in human osteosarcoma cells and tissues. miR-181a-5p mimic significantly promoted, while miR-181a-5p inhibitor blocked the proliferation, colony formation, migration, invasion, and cell cycle progression of osteosarcoma cells. Mechanistically, miR-181a-5p bound to the 3′-untranslational region of phosphatase and tensin homolog (PTEN) and reduced its protein expression, thereby activating protein kinase B (PKB/AKT) pathway. Either PTEN overexpression or AKT inhibition notably blocked the tumor-promoting effects of miR-181a-5p. Moreover, we observed that miR-181a-5p mimic further inhibited growth of human osteosarcoma cells in the presence of adriamycin or cisplatin. Overall, miR-181a-5p promotes osteosarcoma progression via PTEN/AKT pathway and it is a promising therapeutic target to treat osteosarcoma.

## 1. Introduction

Osteosarcoma is the most common primary malignant bone tumor in children and adolescents and contributes to 8.9% of cancer mortality among children [1–3]. Despite the achievements in diagnosis and treatment, the prognosis of osteosarcoma patients remains unsatisfactory with a 5-year overall survival rates no more than 30%. In addition, approximately 40% clinical cases develop rapidly and suffer from recurrence and/or metastasis with fewer available treatments [4–7]. Therefore, it is greatly needed to find novel molecular targets and subsequently provide effective therapeutic strategies for the management of osteosarcoma.

Protein kinase B (PKB/AKT), a conserved serine/threonine protein kinase, is a nodal signaling effector for cell survival and proliferation and serves as an important therapeutic target for osteosarcoma [8, 9]. Emerging studies have validated that AKT activation is sufficient to promote the growth, metastasis, and tumor-mediated bone destruction of osteosarcoma [10–12]. In addition, Shen et al. revealed that AKT activation by long noncoding RNA lncARSR also conferred chemoresistance to adriamycin and facilitated osteosarcoma progression [13]. Conversely, AKT inhibition significantly suppressed osteosarcoma cell proliferation and the malignant phenotype [14, 15]. It is well-accepted that AKT is primarily dephosphorylated and inactivated by phosphatase and tensin homolog deleted on chromosome ten (PTEN), a major tumor suppressor gene in humans [16–18]. Moreover, inhibiting PTEN activity was capable of promoting osteosarcoma progression through activating AKT pathway [19]. Based on these findings, it is reasonable to target PTEN/AKT pathway for the treatment of osteosarcoma.

MicroRNAs (miRNAs) are a class of short noncoding RNAs that post-transcriptionally regulate gene expression through binding to the 3′-untranslated region (UTR) of target mRNAs [20–22]. Numerous miRNAs have been reported to be implicated in the progression of osteosarcoma and accurately predict the long-term outcome of osteosarcoma patients. Zhu et al. found that miR-23b-3p was significantly upregulated in osteosarcoma cells and that knockdown of miR-23b-3p shifted glycolysis to oxidative phosphorylation of osteosarcoma cells, thereby inhibiting osteosarcoma cell proliferation [23]. Results from Liu et al. indicated that miR-210-5p was upregulated in clinical osteosarcoma specimens and cell lines and that miR-210-5p overexpression facilitated epithelial-mesenchymal transition and oncogenic autophagy, causing osteosarcoma overgrowth and pulmonary metastasis [24]. miR-181a-5p plays critical roles in regulating cell survival and proliferation, and aberrant miR-181a-5p expression has been linked with the progression of multiple cancers [25–27]. In addition, Xia et al. determined that miR-181a-5p expression in peripheral blood and cartilage tissues was decreased in osteoarthritis patients and that the inhibition of miR-181a-5p significantly increased tumor necrosis factor *α* and aggravated osteoarthritis [28]. In the present study, we aim to investigate the function and potential molecular basis of miR-181a-5p in osteosarcoma.

## 2. Materials and Methods

### 2.1. Cell Cultures and Treatments

The human osteosarcoma cells SaOS2, MG63, HOS, 143B and U2OS, and a human osteoblast cell hFOB1.19 were purchased from Type Culture Collection of the Chinese Academy of Sciences (Shanghai, China) and cultured in DMEM medium containing 10% fetal bovine serum (FBS) and 1% penicillin/streptomycin at 37°C in a 5% CO_2_ incubator. Cells were incubated with the mimic, inhibitor of miR-181a-5p, or their negative control (NC) at a concentration of 100 nmol/L for 24 h using Lipofectamine 6000 reagent (Beyotime; Shanghai, China), and then cultured in fresh medium supplemented with 10% FBS for additional 96 h [29]. miR-181a-5p mimic (miR10000256-1-5), inhibitor (miR20000256-1-5), and the NC were all purchased from Guangzhou RiboBio Co., Ltd. (Guangzhou, China). To clarify the role of PTEN, cells were infected with adenovirus carrying the full-length PTEN (Ad-PTEN) or green fluorescent protein (Ad-GFP) at a multiplicity of infection of 30 for 4 h and maintained in fresh medium for additional 24 h before miR-181a-5p mimic treatment. The Ad-PTEN and Ad-GFP were all generated by Hanbio Biotechnology Co., Ltd. (Shanghai, China). To inhibit AKT, cells were treated with AKT kinase inhibitor (1 *μ*mol/L) for 24 h before miR-181a-5p mimic treatment [30]. To investigate whether miR-181a-5p mimic treatment would further inhibit growth of osteosarcoma cells in the presence of chemotherapeutic reagents, cells were pretreated with 0.4 *μ*mol/L adriamycin (ADR; Sigma-Aldrich, St. Louis, MO, USA) and 1 *μ*mol/L cisplatin (DDP, Sigma-Aldrich) for 24 h before miR-181a-5p mimic treatment according to previous studies [31, 32].

### 2.2. Cell Viability and Colony Formation Assay

Cell viability was determined using cell count kit-8 (CCK-8) kit (Beyotime, Shanghai, China) [33–36]. Briefly, cells were incubated in serum-free DMEM medium containing 10 *μ*L CCK-8 solution at 37°C for 2 h in the dark, and then the optical density (OD) value was detected at 450 nm using a microplate reader (Synergy HT, BioTek). To detect the colony formation, cells were treated and then cultured for addition 2 weeks with the medium replaced every 3 days. Next, cells were fixed with 4% paraformaldehyde at room temperature for 20 min and stained with 0.2% crystal violet (Wuhan Servicebio Technology Co., Ltd., Wuhan, China) at room temperature for 30 min, followed by the rinse under tap water for additional 20 min. Finally, colonies with the diameter >0.05 mm were counted in a blinded manner [37–39].

### 2.3. Wound Healing and Transwell Experiments

Wound healing experiment was performed to evaluate the migrative capacity of osteosarcoma cells in vitro according to previous studies [40, 41]. In brief, cells were seeded to 6-well plates and allowed to grow to ~90% confluence overnight. Then, a 200 *μ*L yellow pipette tip was used to scratch a gap on the cellular monolayers, and cells were then washed with phosphate buffer (PBS) to remove the suspended cells, followed by the incubation in serum-free DMEM medium for additional 24 h. Images were captured using an optical microscope and the gap sized was recorded. To determine the invasive capacity of osteosarcoma cells, transwell experiment was performed as previously described [42–44]. Briefly, cells at a density of 2 × 10^5^ per well were seeded onto the upper chambers that were precoated with polymerized matrigel, and the lower chambers were filled with 800 *μ*L DMEM medium containing 20% FBS as a chemotactic factor. 48 h later, cells in the upper surfaces were removed and cells in the bottom of the filters were stained with 0.2% crystal violet at room temperature for 30 min. The invasive cells were then counted in a blinded manner.

### 2.4. Cell Cycle Analysis

Cell cycle distribution was analyzed by a flow cytometry using propidium iodide (PI) staining method [45, 46]. Briefly, cells were stained with 0.5 mL PI/RNase Staining Buffer (BD Bioscience, Franklin, NJ, USA) at 37°C for 15 min after being fixed in 70% ethanol at −20°C overnight, and then were subjected to flow cytometry analysis to detect the fractions of the cells in G0/G1, S, and G2/M phase using FACSCalibur flow cytometer (BD Bioscience).

### 2.5. Western Blot

Cells were washed by PBS for 3 times and then lysed in RIPA lysis buffer (Wuhan Servicebio Technology Co., Ltd.) with the protein concentration measured by a Pierce™ Microplate BCA Protein Assay Kit (Invitrogen, Carlsbad, CA, USA) [47–49]. Next, equal amounts of total proteins were separated by sodium dodecyl sulfate-polyacrylamide gel electrophoresis and then transferred onto polyvinylidene fluoride membrane and followed by the incubation in 5% skimmed milk at room temperature for additional 1 h to block the nonspecific binding of primary antibodies. After that, the membranes were incubated with indicated primary antibodies at 4°C overnight and the secondary antibody at room temperature for additional 1 h on the second day. Finally, the bands were visualized using electrochemiluminescence reagent and analyzed by Image Lab software (Version 6.0, Bio-Rad). The primary antibodies against phospho-KT (p-AKT), total-AKT (t-AKT), PTEN, and glyceraldehyde-3-phosphate dehydrogenase (GAPDH) were all purchased from Cell Signaling Technology (Danvers, MA, USA).

### 2.6. Reverse Transcription-Quantitative Polymerase Chain Reaction (RT-qPCR)

The total RNA was extracted from cultured cells with TRIzol reagent (Invitrogen) and reversely transcribed to cDNA using the TaqMan® microRNA Reverse Transcription Kit (Applied Biosystems, Foster City, CA, USA) according to the manufacturer's instructions [50–52]. Then, the relative levels of miR-181a-5p were determined with the TaqMan microRNA Assays Kit (Applied Biosystems) and normalized to U6 using the 2^−*ΔΔ*Ct^ method on a 2.1 Real-Time PCR System using Bio-Rad CFX Manager (Bio-Rad, USA). The sequences were provided as below: miR-181a-5p, forward, 5′-TAGAGCTAGCGAATTCTTTGTTGGAAGAATCATGCTTCT-3′, reverse, 5′-TTGCGGCCGCGGATCCCATTGTTCAGTGAGCTTGTCCAC-3′; U6, forward, 5′-GCTTCGGCAGCACATATACTAAAAT-3′, reverse, 5′-CGCTTCACGAATTTGCGTGTCAT-3′.

### 2.7. Luciferase Reporter Assay

The direct binding of miR-181a-5p to the 3′-UTR of PTEN was validated by the luciferase reporter assay as described previously [53, 54]. In brief, the wild-type (WT) PTEN 3′-UTR with position 2310-2316 or truncated (TNC) PTEN 3′-UTR without position 2310-2316 was cloned into the sites 3′ of the firefly luciferase gene (luc2) of pmirGLO luciferase reporter plasmids (Promega Corporation, Madison, WI, USA), which were then cotransfected with miR-181a-5p mimic or mimic-NC to the HEK293T cells using Lipofectamine 6000 reagent (Beyotime). 48 h later, the fluorescent changes were measured using the dual luciferase reporter assay system with the firefly luciferase activity normalized to the corresponding Renilla luciferase activity.

### 2.8. Human Samples

Human osteosarcoma and adjacent normal tissues were obtained from 96 osteosarcoma patients who did not undergo radiotherapy or chemotherapy before surgery, and their clinicopathological characteristics were provided in supplementary Table. All tissues were preserved in liquid nitrogen immediately after resection and stored at −80°C before further detection. Written informed consent was obtained from the patients or their legal guardians in accordance with the guidelines of in the Declaration of Helsinki. All experimental procedures were approved by the institutional review board and the ethics committee of our hospitals (Approval No. syrmyy2019-174).

### 2.9. Statistical Analysis

The SPSS software (Version 22.0) was used for statistical analysis with the *P* < 0.05 considered statistically significant. All data were presented as the mean ± SD, and unpaired Student's *t*-test test and one-way ANOVA followed by Tukey's post hoc test were used for comparisons between 2 groups and among 3 or more groups.

## 3. Results

### 3.1. miR-181a-5p Level is Elevated in Human Osteosarcoma Cells and Tissues

**Figure 1:**
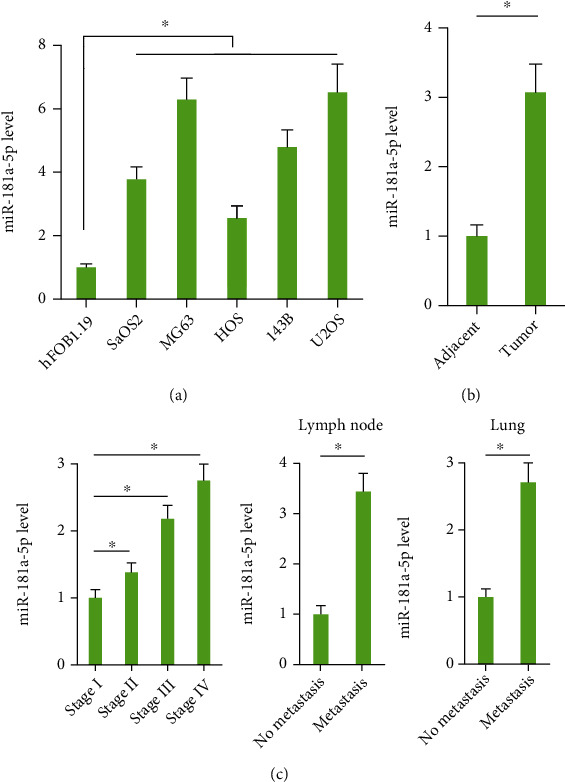
miR-181a-5p level is elevated in human osteosarcoma cells and tissues. (a) Relative miR-181a-5p level in different osteosarcoma cells and healthy human osteoblast cell (*n* = 6). (b) Relative miR-181a-5p level in human osteosarcoma and adjacent normal samples (*n* = 96). (c) Relative miR-181a-5p level in human osteosarcoma samples was analyzed according to the tumor stage and metastasis status (*n* = 12). All data are presented as the mean ± SD, ^∗^*P* < 0.05 versus the matched group.

First, we detected the level of miR-181a-5p in six human osteosarcoma cell lines and a human osteoblast cell hFOB1.19. As shown in Figure 1(a), miR-181a-5p level was significantly elevated in human osteosarcoma cells, especially the MG63 and U2OS cells. We then examined the expression of miR-181a-5p in 24 paired osteosarcoma tissues and adjacent normal tissues and detected a remarkable elevation of miR-181a-5p in human osteosarcoma tissues compared with healthy tissues (Figure 1(b)). In addition, increased miR-181a-5p level positively correlated with the advanced TNM stage, lymph node, and lung metastasis in osteosarcoma patients (Figure 1(c)). Together, these data indicate that miR-181a-5p may be implicated in the progression of human osteosarcoma.

### 3.2. Mir-181a-5p Inhibitor Restrains Osteosarcoma Progression In Vitro

**Figure 2:**
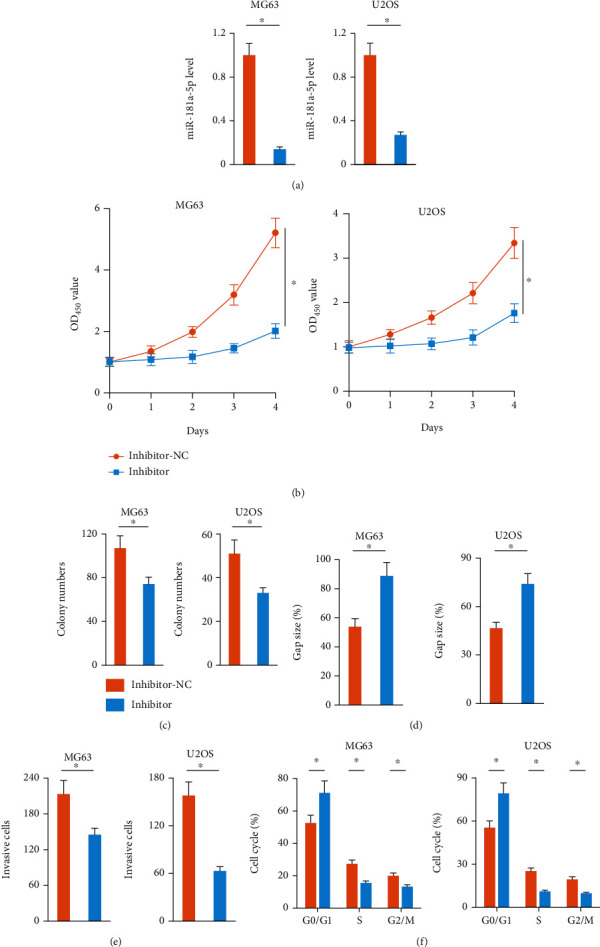
miR-181a-5p inhibitor restrains osteosarcoma progression in vitro. (a) Relative miR-181a-5p level in osteosarcoma cells treated with or without miR-181a-5p inhibitor (*n* = 6). (b) Cell viability of osteosarcoma cells determined by CCK-8 assay (*n* = 6). (c) Colony numbers in osteosarcoma cells with or without miR-181a-5p inhibitor treatment (*n* = 6). (d) Gap size in the wound healing experiments (*n* = 5). (e) Invasive cells in transwell experiments with or without miR-181a-5p inhibitor treatment (*n* = 6). (f) Cell cycle distribution in different phases (*n* = 5). All data are presented as the mean ± SD, ^∗^*P* < 0.05 versus the matched group.

In the above study, MG63 and U2OS cells were found to have higher miR-181a-5p expression than other osteosarcoma cells; therefore, we used these two cells in our further study. To clarify the role of miR-181a-5p in osteosarcoma cells, MG63 and U2OS cells were transfected with miR-181a-5p inhibitor to reduce the expression of endogenous miR-181a-5p (Figure 2(a)). As determined by the CCK-8 assay, we found that miR-181a-5p inhibitor significantly reduced human osteosarcoma cell viability in vitro (Figure 2(b)). As shown in Figure 2(c), the cells treated with miR-181a-5p inhibitor also had decreased colony formative capacity. The above-mentioned study (Figure 1(c)) showed that miR-181a-5p level positively correlated with the advanced TNM stage, lymph node, and lung metastasis in osteosarcoma patients. Migration and invasion are two determinants of the malignant osteosarcoma phenotype and are associated with the local bone destruction and distant metastasis [55, 56]. Wound-healing and transwell experiments were performed to evaluate the migrative and invasive capacities of osteosarcoma cells. As expected, miR-181a-5p inhibitor significantly suppressed MG63 and U2OS cells migration and invasion (Figures 2(d) and 2(e)). In addition, the percentage of G1/G0 phase cells was increased, while the percentage of S and G2/M phase cells was decreased in miR-181a-5p inhibitor-treated osteosarcoma cells (Figure 2(f)). Collectively, these findings suggest that miR-181a-5p inhibitor suppresses the growth, colony formation, migration and invasion of osteosarcoma cells in vitro, and induces G1/G0 cell cycle arrest.

### 3.3. Mir-181a-5p Mimic Promotes Osteosarcoma Progression In Vitro

**Figure 3:**
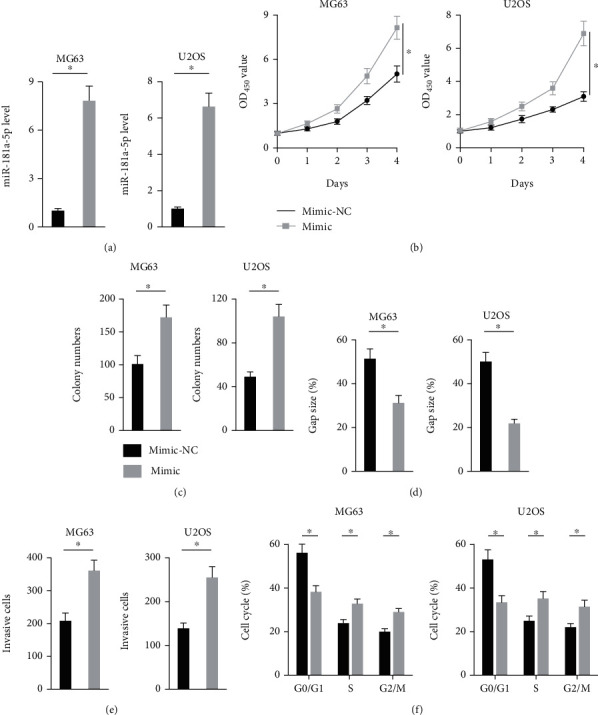
miR-181a-5p mimic promotes osteosarcoma progression in vitro. (a) Relative miR-181a-5p level in osteosarcoma cells treated with or without miR-181a-5p mimic (*n* = 6). (b) Cell viability of osteosarcoma cells determined by CCK-8 assay (*n* = 6). (c) Colony numbers in osteosarcoma cells with or without miR-181a-5p mimic treatment (*n* = 6). (d) Gap size in the wound healing experiments (*n* =5). (e) Invasive cells in transwell experiments with or without miR-181a-5p mimic treatment (*n* = 6). (f) Cell cycle distribution in different phases (*n* = 5). All data are presented as the mean ± SD, ^∗^*P* < 0.05 versus the matched group.

Then, MG63 and U2OS cells were treated with miR-181a-5p mimic to investigate whether overexpression could promote osteosarcoma progression in vitro (Figure 3(a)). As shown in Figures 3(b) and 3(c), miR-181a-5p mimic significantly increased MG63 and U2OS cells viability and colony formation. And their migrative and invasive capacities were also enhanced by miR-181a-5p mimic treatment, indicating an increased malignance of the MG63 and U2OS cells (Figures 3(d) and 3(e)). In addition, miR-181a-5p mimic also significantly decreased the cell numbers in G0/G1 phase yet increased the cells in S and G2/M phase (Figure 3(f)). In addition, we also examined the role of miR-181a-5p mimic in HOS cell with low expression of miR-181a-5p. As shown in Supplementary Figure 1A–1E, miR-181a-5p mimic significantly promoted the proliferation, colony formation, migration ,and invasion of HOS cell in vitro. These data reveal that miR-181a-5p mimic contributes to the proliferation, colony formation, migration, invasion, and cell cycle progression of osteosarcoma cells in vitro.

### 3.4. Mir-181a-5p Modulates Osteosarcoma Progression via PTEN-AKT Pathway In Vitro

**Figure 4:**
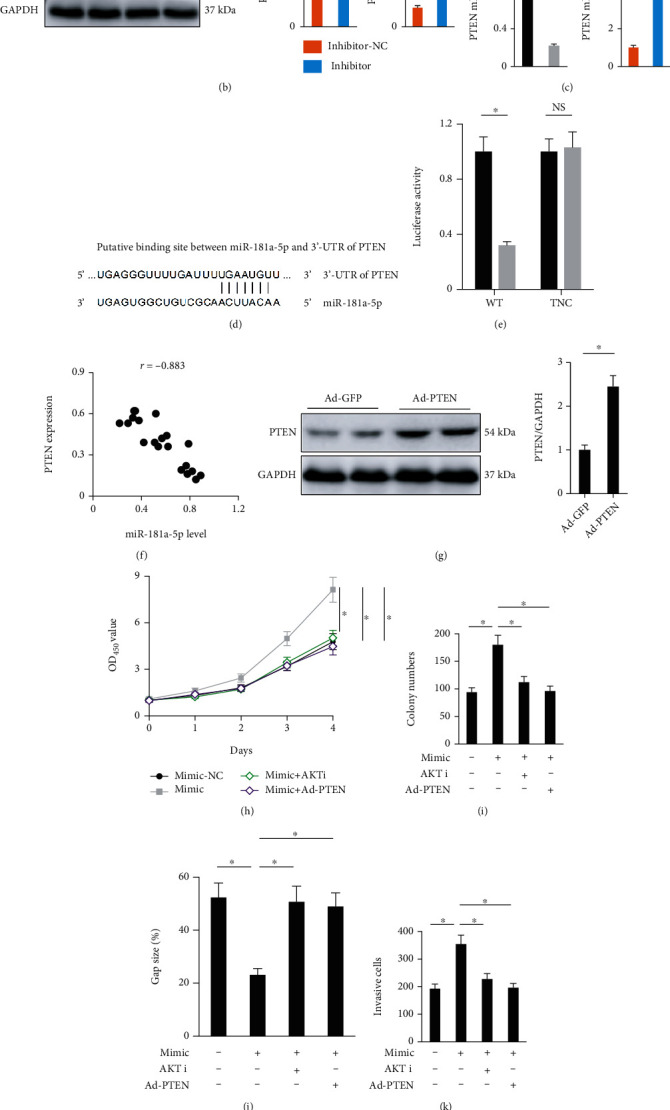
miR-181a-5p modulates osteosarcoma progression via PTEN-AKT pathway in vitro. (a and b) Representative western blot images and the statistical analysis of AKT and PTEN (*n* = 6). (c) Relative PTEN mRNA levels in osteosarcoma cells treated with miR-181a-5p mimic or inhibitor (*n* = 6). (d) Putative binding site of between miR-181a-5p and 3′-UTR of PTEN. (e) Relative luciferase activity in HEK293T cells (*n* = 6). (f) Correlation between miR-181a-5p level and PTEN expression in human osteosarcoma tissues (*n* = 20). (g) Representative western blot images and the statistical analysis of PTEN (*n* = 6). (h and i) Cell viability and colony numbers in osteosarcoma cells (*n* = 6). (j and k) Gap size and invasive cells in osteosarcoma cells (*n* = 6). (l) Cell cycle distribution in different phases (*n* = 5). All data are presented as the mean ± SD, ^∗^*P* < 0.05 versus the matched group. (l) ^∗^*P* < 0.05 versus control group, ^#^*P* < 0.05 versus miR-181a-5p mimic-treated osteosarcoma cells.

AKT is a nodal signaling effector for cell survival and proliferation and serves as an important therapeutic target for osteosarcoma [14, 15]. We then investigated whether miR-181a-5p modulates osteosarcoma progression through AKT. As shown in Figures 4(a) and 4(b), AKT in MG63 cells was activated by miR-181a-5p mimic yet inhibited by miR-181a-5p inhibitor. PTEN is a major tumor suppressor gene in humans and functions as a classic upstream phosphatase of AKT pathway. Intriguingly, we found that PTEN mRNA and protein levels were decreased in miR-181a-5p mimic-treated MG63 cells, but increased in miR-181a-5p inhibitor-treated MG63 cells (Figures 4(a)–4(c)). In addition, a putative binding site of miR-181a-5p was observed in the 3′-UTR of PTEN using the TargetScan online software (Figure 4(d)). To validate the prediction, luciferase reporter assay was performed. The data suggested that miR-181a-5p mimic could directly inhibit the luciferase activity in HEK293T cells transfected with WT 3′-UTR of PTEN but not the TNC form (Figure 4(e)). Meanwhile, we observed a negative correlation between miR-181a-5p level and PTEN expression in human osteosarcoma tissues (Figure 4(f)). Next, we overexpressed PTEN in MG63 cells with the adenovirus and the efficiency was validated by the western blot (Figure 4(g)). As shown in Figures 4(h) and 4(i), miR-181a-5p mimic significantly increased the viability and colony formation in MG63 cells, which were almost completely abolished by either PTEN overexpression or AKT inhibition. And the increased migrative and invasive capacities seen in miR-181a-5p mimic-treated MG63 cells were also decreased by either PTEN overexpression or AKT inhibition (Figures 4(j) and 4(k)). In addition, both PTEN overexpression and AKT inhibition could significantly blocked miR-181a-5p mimic-mediated cell cycle progression in MG63 cells (Figure 4(l)). Taken together, our findings identify that miR-181a-5p modulates osteosarcoma progression via PTEN-AKT pathway in vitro.

### 3.5. Mir-181a-5p Inhibitor Further Inhibits Growth of Osteosarcoma In Vitro in the Presence of Classic Chemotherapeutic Agents

**Figure 5:**
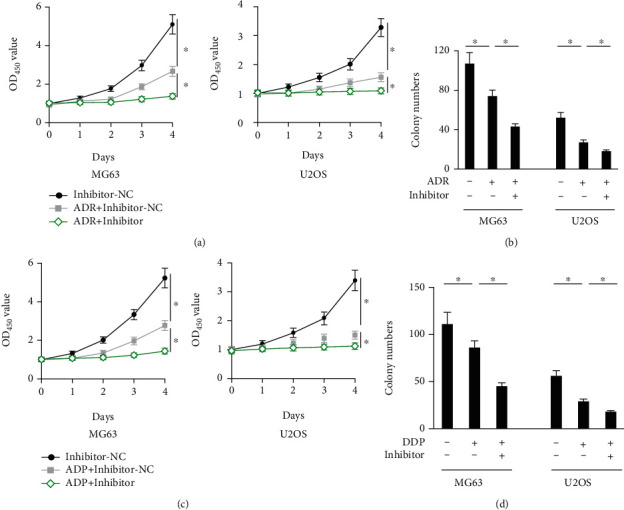
miR-181a-5p inhibitor further inhibits growth of osteosarcoma in vitro in the presence of classic chemotherapeutic agents. (a) Cell viability of osteosarcoma cells determined by CCK-8 assay (*n* = 6). (b) Colony numbers in ADR-treated osteosarcoma cells with or without miR-181a-5p inhibitor incubation (*n* = 6). (c) Cell viability of osteosarcoma cells determined by CCK-8 assay (*n* = 6). (d) Colony numbers in DDP-treated osteosarcoma cells with or without miR-181a-5p inhibitor incubation (*n* = 6). All data are presented as the mean ± SD, ^∗^*P* < 0.05 versus the matched group.

Since miR-181a-5p inhibitor significantly restrained osteosarcoma progression in vitro, we then determined whether miR-181a-5p inhibitor could further inhibit growth of human osteosarcoma cells in the presence of ADR or DDP, two common chemotherapeutic agents for the treatment of osteosarcoma. As shown in Figures 5(a) and 5(b), ADR incubation significantly inhibited the viability and colony formation in human osteosarcoma cells, which were further enhanced in the presence of miR-181a-5p inhibitor. As expected, miR-181a-5p inhibitor also further inhibited growth of DDP-treated MG63 and U2OS cells, as determined by the decreased cell viability and colony formation (Figures 5(c) and 5(d)). Collectively, the results approve that miR-181a-5p inhibitor can further inhibit growth of osteosarcoma in vitro in the presence of classic chemotherapeutic agents, at least ADR and DDP.

## 4. Discussion

Osteosarcoma is identified as a malignant bone tumor in adolescents with a poor prognosis due to pulmonary metastasis. Given the deeper understanding of the pathophysiological mechanism and rapid progress of medical treatment for osteosarcoma, the 5-year survival rate in nonmetastatic patients has reached from 20% to 60%–70% in the past 30 years; however, approximately 40% clinical cases suffer from recurrence and/or metastasis with very low survival rate [2, 3, 7]. In the present study, we reveal that miR-181a-5p level is elevated in human osteosarcoma cells and tissues, and positively correlates with the advanced TNM stage, lymph node, and lung metastasis in clinical cases. miR-181a-5p mimic contributes to, while miR-181a-5p inhibitor suppresses the proliferation, colony formation, migration, invasion, and cell cycle progression of osteosarcoma cells in vitro through regulating PTEN-AKT pathway. More importantly, miR-181a-5p inhibitor can further inhibit growth of human osteosarcoma cells in the presence of chemotherapeutic agents (e.g. ADR and DDP). Collectively, our data determine that the increased miR-181a-5p level in osteosarcoma is essential for its development and the malignant potential, and that inhibiting miR-181a-5p may provide novel therapeutic strategies to control osteosarcoma.

Emerging evidences have verified that the aberrant miRNAs are implicated in the progression of osteosarcoma via regulating multiple biological processes, including growth, migration, and invasion and cell cycle, miR-181a-5p has been linked with various human cancers, including breast cancer, prostate cancer, and melanoma and non-small-cell lung cancer. Benedetti et al. demonstrated that miR-181a-5p was downregulated in human breast cancer samples and could be used as a promising biomarker for prognosis of primary breast cancer [57]. Results from Wang et al. indicated that serum miR-181a-5p was significantly elevated in prostate cancer patients with bone metastasis than those in non-bone metastatic prostate cancer groups and could predict the risk of bone metastasis for prostate cancer patients [58]. In a previous study, the authors showed that miR-181a-5p directly targeted and inhibited BCL-2, thereby reducing melanoma stem cells apoptosis and promoting the tumorigenesis of melanoma in vivo [59]. In contrast, Li et al. demonstrated that miR-181a-5p suppression by a long noncoding RNA SNHG7 facilitated the proliferation, migration, and invasion of non-small-cell lung cancer [60]. Mutlu and colleagues detected a significant upregulation of miR-181a-5p in human chondrosarcoma and furthermore, Xiong et al. identified a dysregulated miR-181a-5p expression between metastatic and non-metastatic osteosarcoma [61, 62]. However, relative little is known about the role of miR-181a-5p in osteosarcoma progression. Herein, we identified an oncologic role of miR-181a-5p in human osteosarcoma, and miR-181a-5p inhibitor significantly suppressed the proliferation, colony formation, migration, invasion and cell cycle progression, and enhanced the chemosensitivity of osteosarcoma cells in vitro.

AKT, a multifunctional kinase, plays critical roles in regulating cell growth and is constitutively activated in various human cancers, including osteosarcoma [14, 63]. AKT directly phosphorylates and activates mammalian target of rapamycin (mTOR), a crucial signaling nexus to orchestrate protein synthesis and cell growth [64]. Wang et al. demonstrated that AKT-mediated activation of mTOR in osteosarcoma cells significantly upregulated glycolysis-related proteins and migration-related proteins, thereby facilitating osteosarcoma progression [65]. Xiao et al. showed that the activation of AKT-mTOR axis promoted autophagy and led to osteosarcoma chemoresistance [66]. Zhang and colleagues previously validated that AKT activation resulted in increased phosphorylation and nuclear translocation of nuclear factor kappa-B, eventually enhancing epithelial-mesenchymal transition and migration of MG-63 osteosarcoma cells [67]. Under physiological conditions, AKT is dephosphorylated and inactivated by PTEN through the lipid phosphatase activity, a classical tumor suppressor gene in humans [14]. Herein, we disclosed that miR-181a-5p directly bound to the 3′-UTR of PTEN and reduced PTEN protein expression, thereby promoting AKT activation and osteosarcoma progression.

In summary, our data for the first time reveal that miR-181a-5p promotes osteosarcoma progression via PTEN/AKT pathway and that miR-181a-5p is a promising therapeutic target to treat osteosarcoma.

## Data Availability

The data that support the findings of this study are available from the corresponding author upon reasonable request.

## Supplementary Materials

Supplementary Materials Table S1: clinicopathological characteristics of 96 osteosarcoma patients; Figure S1: miR-181a-5p mimic promotes osteosarcoma progression in vitro.
