# Supplementary material for: MicroRNA-181a-5p Promotes Osteosarcoma Progression via PTEN/AKT Pathway
Source: Anal Cell Pathol (Amst). 2022 Mar 8;2022:3421600. doi: 10.1155/2022/3421600 (PMC8924609; doi:10.1155/2022/3421600)
Supplement: Supplementary Materials — Table S1: clinicopathological characteristics of 96 osteosarcoma patients; Figure S1: miR-181a-5p mimic promotes osteosarcoma progression in vitro. [file 3421600.f1.docx]

**Supplementary Table: Clinicopathological characteristics of 96 osteosarcoma patients**

| Variable | n=96 |
| --- | --- |
| Age at surgery  <20  >=20 | 69  27 |
| Gender  Male  Female | 56  40 |
| TNM stage  I  II  III  IV | 18  39  24  15 |
| Anatomic location  Tibia/femur  Elsewhere | 73  23 |
| Lymph node metastasis  Yes  No | 56  40 |
| Lung metastasis  Yes  No | 39  57 |


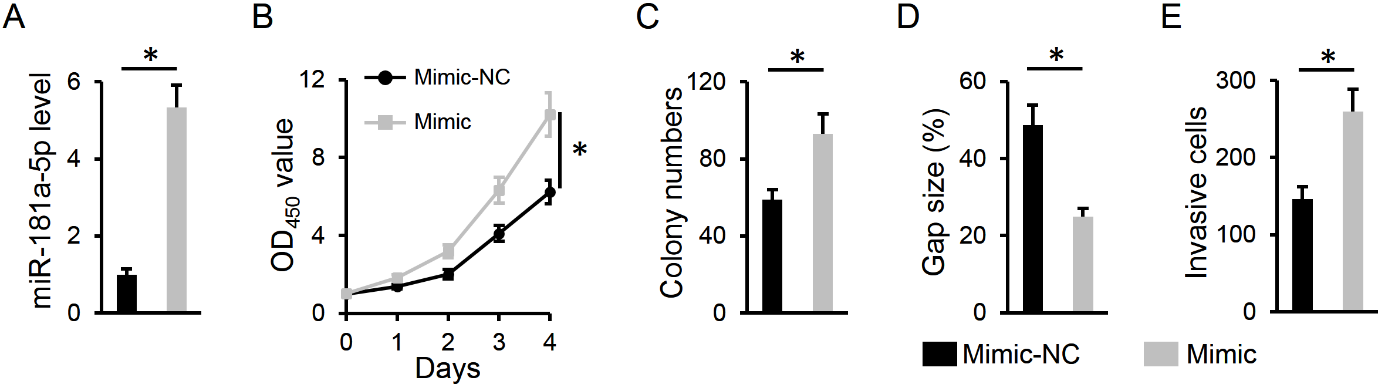


**Supplementary Figure 1. miR-181a-5p mimic promotes osteosarcoma progression in vitro. (A)** Relative miR-181a-5p level in HOS cell treated with or without miR-181a-5p mimic (n=6). **(B)** Cell viability of HOS cell determined by CCK-8 assay (n=6). **(C)** Colony numbers in HOS cell with or without miR-181a-5p mimic treatment (n=6). **(D)** Gap size in the wound healing experiments (n=5). **(E)** Invasive cells in Transwell experiments with or without miR-181a-5p mimic treatment (n=6). All data are presented as the mean ± SD, **P* < 0.05 versus the matched group.
